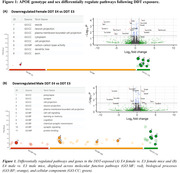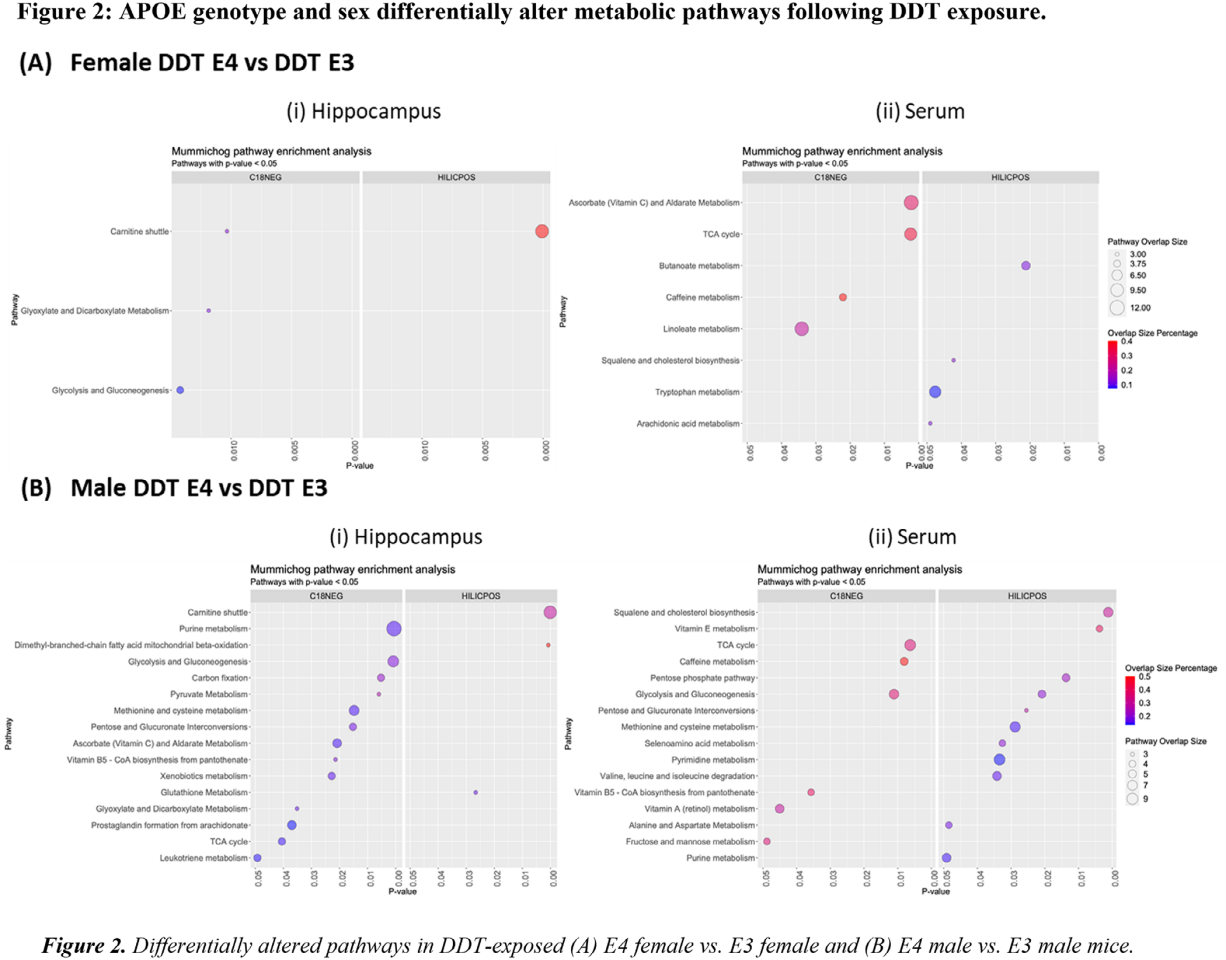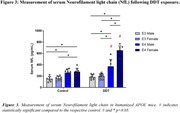# Multi‐omics assessment of genotype and sex‐specific effects of organochlorine pesticide DDT in humanized APOE mice

**DOI:** 10.1002/alz.089489

**Published:** 2025-01-03

**Authors:** Isha Mhatre‐Winters, Aseel Eid, Jiada J Zhan, Ronald P Hart, Arturo J Barahona, ViLinh Tran, Yoonhee Han, Young‐Mi Go, Dean P Jones, Jason R Richardson

**Affiliations:** ^1^ Isakson Center for Neurological Disease Research, College of Veterinary Medicine, University of Georgia, Athens, GA USA; ^2^ Robert Stempel College of Public Health and Social Work, Florida International University, Miami, FL USA; ^3^ Nutrition & Health Sciences Doctoral Program, Laney Graduate School, Emory University, Atlanta, GA USA; ^4^ Department of Cell Biology & Neuroscience, Rutgers University, Piscataway, NJ USA; ^5^ Department of Medicine, Emory University, Atlanta, GA USA

## Abstract

**Background:**

The Apolipoprotein‐E (APOE) ε4 gene variant is the strongest genetic risk factor for late‐onset Alzheimer’s Disease, but is not entirely predictive. Emerging evidence suggests environmental factors contribute to disease etiology, with epidemiological studies associating pesticide exposure with lower cognitive scores. Dichlorodiphenyltrichloroethane (DDT), a pesticide used extensively in the US until 1972, persists in trace amounts due to its long half‐life, bioaccumulation, and existing dumpsites. We have previously reported that DDT induces AD pathology in multiple models and also shown increased serum levels of DDE, the primary metabolite of DDT, in AD patients, where APOE genotype modified MMSE scores and contributed a ∼4‐fold increase in AD risk. Here, we sought to identify the mechanism(s) by which APOE4 genotype alters pathological response to DDT by a multi‐omics approach.

**Method:**

To assess the effects of DDT exposure, three‐month‐old male and female APOE3 (E3) and APOE4 (E4) mice were exposed to 3 mg/kg DDT by oral gavage every 3 days for 5 months. Hippocampus was dissected out and frozen for RNA‐sequencing and metabolomic analysis, while serum was collected for metabolomic analysis and biomarker measurement.

**Result:**

RNA‐sequencing analysis between genotypes of female DDT‐exposed mice indicated significantly downregulated pathways and functions (p<0.05) in the hippocampus of E4‐females, including phosphatidylserine decarboxylase activity and neuron projection. Similarly, DDT‐exposed E4‐males indicated downregulation of ion channel regulator activity, nucleotide binding, cell‐cell and synaptic signaling, and neuron projection. Further, untargeted metabolomic analysis of DDT‐exposed mice was compared. Top pathways (p<0.05) altered in E4 vs. E3 female‐DDT hippocampus were carnitine shuttle and glycolysis‐gluconeogenesis, while serum changes included TCA cycle and tryptophan metabolism. In E4 vs. E3 male‐DDT, hippocampal alterations included purine metabolism, fatty acid mitochondrial beta‐oxidation, TCA cycle, glycolysis, and carnitine shuttle, while serum changes involved cholesterol biosynthesis, TCA cycle, and glycolysis‐gluconeogenesis. Finally, DDT increased serum neurofilament light chain (NfL) levels, a biomarker for neurodegeneration, by 1.9 and 2.1‐fold in E4‐male and E4‐female mice compared to respective controls. NfL was 1.4‐fold higher in DDT‐exposed E4‐females compared to E4‐males.

**Conclusion:**

These data demonstrate gene‐by‐environment interactions modified by sex, providing a platform to investigate mechanisms in AD‐related neurodegeneration and corroborating previous epidemiological findings.